# Evaluation of User-Prosthesis-Interfaces for sEMG-Based Multifunctional Prosthetic Hands

**DOI:** 10.3390/s21217088

**Published:** 2021-10-26

**Authors:** Julio Fajardo, Guillermo Maldonado, Diego Cardona, Victor Ferman, Eric Rohmer

**Affiliations:** 1 Turing Research Laboratory, FISICC, Galileo University, Guatemala City 01010, Guatemala; guiller@galileo.edu (G.M.); juandiego.cardona@galileo.edu (D.C.); 2 Department of Computer Engineering and Industrial Automation, FEEC, UNICAMP, Campinas 13083-852, Brazil; vferman@dca.fee.unicamp.br (V.F.); eric@dca.fee.unicamp.br (E.R.)

**Keywords:** assistive robotics, upper-limb prosthesis, electromyography, user-prosthesis interface

## Abstract

The complexity of the user interfaces and the operating modes present in numerous assistive devices, such as intelligent prostheses, influence patients to shed them from their daily living activities. A methodology to evaluate how diverse aspects impact the workload evoked when using an upper-limb bionic prosthesis for unilateral transradial amputees is proposed and thus able to determine how user-friendly an interface is. The evaluation process consists of adapting the same 3D-printed terminal device to the different user-prosthesis-interface schemes to facilitate running the tests and avoid any possible bias. Moreover, a study comparing the results gathered by both limb-impaired and healthy subjects was carried out to contrast the subjective opinions of both types of volunteers and determines if their reactions have a significant discrepancy, as done in several other studies.

## 1. Introduction

Several works in the literature present substantial progress in advanced bionic prosthetic devices in recent years, offering people with disabilities many different alternatives and characteristics to improve their condition. This progress includes promising works in haptics [1,2] and diverse methods to recover and interpret the user intent [3,4,5,6]. However, little to no effort has been directed into research for providing a simple and easy-to-use user-prosthesis interface (UPI). This aspect is directly related to the patient’s subjective perception of the prosthetic device itself, greatly influencing its use or not. This way, it has been already proven that the acceptability for such devices depends more on the lack of effort to operate it than consistently achieving successful grasps [7].

Some methods to operate upper-limb prostheses do not implement a graphical UPI, controlling the device exclusively by analyzing a specific activation profile based on processing electromyography (EMG) signals. Some of these iterations substitute the visual stimuli by utilizing other types of feedback, such as vibrotactile ones [7]. Moreover, others include implants that utilize Bluetooth or radio channel waves to communicate with them [3,8,9]. These versions use wireless charging to function and regulate the power dissipation inside a safe range to avoid damage to the user’s skin tissue.

On the other hand, some approaches use brain-machine interfaces (BMI) to control these devices, eliminating any visual stimulus to interact with the artificial limb and resembling the way limbs are usually operated. Newer methodologies are based on high-density electrocorticography (ECoG), which allows the patient to control each finger individually through an adequate re-innervation process [4]. However, these interfaces require very intrusive and expensive procedures. Other projects utilize interaction processes that do not seem intuitive to the users, employing more creative approaches to analyze the EMG signals by using other members to drive the movements of the prosthetic limb, as shown in [5,6], which use the toes and the tongue, respectively. Such techniques result in viable alternatives, especially for bilateral amputees. However, such methodologies may not be the best option for unilateral transradial amputees since they affect how some typical activities of daily living (ADLs) must be carried out.

Alternatively, the majority of sophisticated research assistive devices are based on multimodal approaches. These methodologies usually consist of taking a set of predefined and well-known EMG features and complementing them with information from other kinds of sensors such as inertial measurement units (IMUs), micro-electromechanical systems (MEMS) microphone, mechanomyography (MMG), or force myography (FMG) showing a substantial improvement in classification rates and bi-manual performance [10,11,12,13]. This approach has been used successfully to improve the user control of prosthetic devices in different manners, such as using a multimodal system with Radio Frequency Identification (RFID) tags on specific objects. In this stance, the cognitive effort is reduced to operate an upper-limb prosthetic device and address some of the well-known issues of EMG techniques, such as the limb position effect [14,15,16]. Other stances have been taken into account using the multimodal approach, such as utilizing voice-control, in tandem with visual feedback through a small embedded touchscreen LCD, providing the users with other alternatives to control their prosthetic device in different manners [17,18].

Finally, other studies have been carried out to increase upper-limb prostheses’ functionality, combining surface EMG (sEMG) and deep-learning-based artificial vision systems. This approach works by associating a subset of predefined objects to a list of specific grasps based on the target’s geometric properties, which are gathered by different types of cameras. Such classification processes are fulfilled via convolutional neural networks (CNN) employing customized image object classifiers.

This work focuses on a methodology to evaluate how different UPIs for transradial upper-limb prostheses influence the user’s workload and how user-friendly they are. It is known that several studies have been conducted to evaluate specific prosthetic devices with unimpaired subjects only [19,20,21,22]. The evaluation is subjective, and some assumptions are made regarding the limb-impaired that may not always be accurate. Therefore, these evaluation processes may show a practical and moral dilemma, especially true when considering the interaction process with assistive devices. Therefore, an extension of previous works [22,23] was carried out, in which the results of the evaluation process were collected only with unimpaired subjects. This work includes results of an evaluation process from information gathered from impaired ones and compares results of both types. Thus, we verify that the results obtained from both are strongly related and verify the viability and validity of creating such supposition.

The evaluation process was achieved by employing a customized EMG wireless module (Thalmic Labs’ Myo armband ) to gather user intent, facilitating the device’s installation independently of the user, and then comparing the retrieved results on the impact that certain aspects may have on the interaction process. The module was selected for operating the different UPIs through this work since it is an affordable and viable replacement for the medical-grade sensors (process and classifies sEMG signals by itself), even with subjects with different levels of transradial amputation [4,24,25,26]. Its small subset of self-classified contractions can be adapted to perform a greater number of gestures and grips. These features facilitate its utilization and the replication of all the interfaces since its installation process is more comfortable than wired alternatives or implants, removing any possible bias regarding the sensors to gather the users’ intent evaluating only the UPIs. In this way, the NASA Task Load Index (TLX) scale was employed to estimate the workload evoked from considered UPIs for its evaluation. Besides, a survey describing the UPI’s user-friendliness was perceived and compared the results using a multifactorial ANOVA analysis in order to determine how user-friendly an interface is.

The rest of this work is structured as follows: Section 2 elaborates on the state of the art of the existing methods to evaluate UPIs. Section 3 describes how the whole system is integrated and elaborates on the details of the replicated UPIs for its evaluation. Section 4 describes the evaluation processes and their interpretations. Finally, the last section, Section 5, deals with the impact of the results.

## 2. State of the Art

Since the development of UPIs has not been a focus in commercial or academic works, the ones that center themselves in analyzing the interaction between the user and the artificial limb are also scarce and usually focus on gathering the user intent, such as comparing the efficiency of EMG methods with force, position, tactile, or even joystick controls [27,28]. Nevertheless, most of these results conclude a non-significant difference between them or the EMG one’s superiority. Other methodologies achieve enhancements to collect that information by using hybrid systems, such as using near-infrared spectroscopy (NIRS) [29], or like the ones juxtaposed in [30]. On the other hand, works like [31] delve into the impact of short-term adaptation with independent finger position control and the relevance of the real-time performance of the prosthetic control and its “offline analyses”.

Nonetheless, none of the previously mentioned studies provide details on assessing interfaces in terms of how to interact with the artificial limb with the selected control. However, some works have centered on comparing two primary interfaces, pattern recognition (PR), and direct control [32,33,34]. Some of them even considered active users’ subjective opinions and the objective ones from therapists for a perception analysis on multi-functional upper-limb prostheses [35]. This resulted in general disapproval for the conventional control for switching between actions and the unreliability of the pattern recognition algorithm altogether (even though its speed was praised). Nonetheless, a similar approach has not been taken for a more extensive array of interfaces (at the best of the authors’ knowledge).

Furthermore, regarding the tools that can be used to evaluate assistive robotics, one can find the Psychosocial Impact of Assistive Devices Scale (PIADS), whose purpose is “to assess the effects of an assistive device on functional independence, well-being, and quality of life”. This reflects the self-described experience of the users and may provide insight on the long-term use or disuse [36]. Another method that has been utilized to evaluate assistive robotics is the use of the Human Activity Assistive Technology (HAAT) model, an outline of clinically relevant aspects that need to be considered in the practice. This method provides “enhanced access and application for occupational therapists, but poses challenges to clarity among concepts” [37]. In addition to those, the Southampton Hand Assessment Procedure (SHAP) also helps to identify which grips are better suited for specific prosthetic designs, as it was created to measure the operating range of a hand. However, it has been criticized for some inconsistencies during the assessment of artificial hands and the lack of a measure for their efficiency [38]. Another tool commonly employed is the NASA Task Load Index scale, used to derive an estimate of the workload of different types of tasks and simulations [39]. Its implementation has been, mostly, centered on quantifying the subjective perception of interface designs [40], some of them involving assistive robotics [11,19].

## 3. Materials and Methods

### 3.1. Galileo Hand

**Figure 1:**
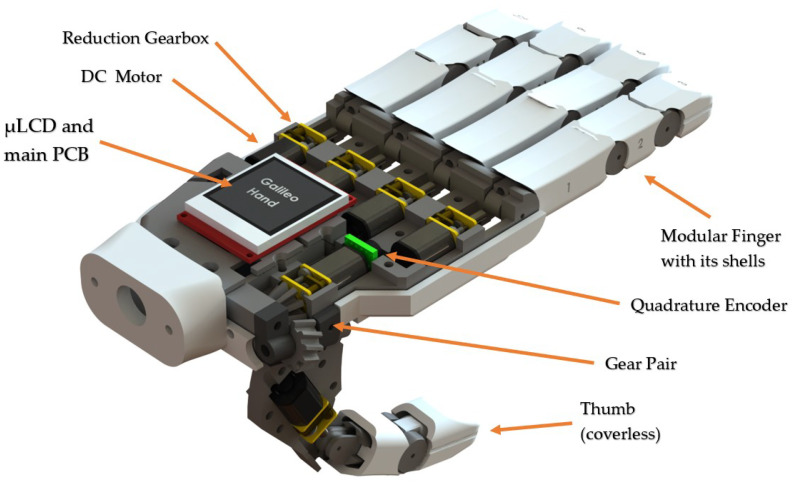
Galileo Hand: anthropomorphic, 3D-printed upper-limb prosthesis.

The Galileo Hand (shown in Figure 1) was the prosthetic device selected to validate this work. This prosthesis is an open-source and intrinsic device that encases five metal-geared micro DC motors to drive the under-tendon-driven (UTD) [41,42] mechanism of each finger, plus an additional DC motor with a quadrature encoder attached to perform the thumb rotation. This device consists of an anthropomorphic, modular, and intrinsic 3D-printed ABS shell; its weight and fabrication cost are under 350 g and USD 350, respectively. Its main controller PCB is based on the ARM Cortex-M4 microcontroller unit (MCU), consisting of the PRJC Teensy 3.2 development board in tandem with three TI DRV8833 dual motor drivers and one 4D-Systems’ 1.44 μLCD-144-G2 screen used to present visual feedback from the UPIs to the users [18,23].

Each finger is assembled using waxed strings, which, when are coiling, close the fingers individually. This process is achieved by motors installed on each finger, providing 5 degrees of actuation (DOA), plus an additional one for the thumb’s rotation. These mechanisms are also made up of surgical-grade elastics that allow the fingers’ articulations to spring back open in a UTD machine model. This configuration provides a total of 15 degrees of freedom (DOF), 1 for the rotation of the tumb and 14 comprised by each joint in the fingers to simulate flexion and extension (three for each digit, except for the thumb, which only has two links and two joints). In addition, the thumb is at a 15${}^{\circ }$ angle from the palmar to emulate both adduction-abduction and opposition-deposition finger movements.

### 3.2. Software

#### 3.2.1. Adapting the Myo Armband

**Figure 2:**
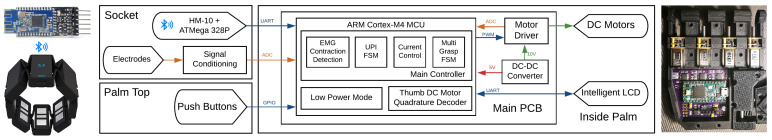
System block diagram showing the embedded controller architecture and the integration with external modules.

Since the proposed solution is to incorporate the Myo armband to capture the muscle’s processed electric signals, a Bluetooth Low Energy (BLE) module, HM-10, was required to transmit them to the Galileo Hand as interpreted poses. Utilizing the MyoBridge library and adapting the hardware according to what was proposed in [43] allows for a successful exchange between the components. The gathered information is later transferred to an ATmega328P (secondary microcontroller unit) and, posteriorly, to the main MCU to drive each DC motor; this is illustrated in Figure 2.

The complementary MCU is in charge of acquiring the user intent, either as raw EMG signals or as Myo-specific poses. Consequently, it converts them into packages transmitted via UART to the Galileo Hand’s central controller. The HM-10’s firmware was flashed with the MyoBridge program, using RedBearLab’s CCLoader as an aide for this procedure to function aptly. This way, the armband will be able to connect with the BLE module and transmit the EMG signals correctly. This process was carried out for most of the interfaces, except for the one using an Android app, since the Myo can be connected, by default, directly to the mobile device.

#### 3.2.2. System Integration

Packet reception is handled using UART interruptions. Once the package is received, it is evaluated, and action is taken based on the content of the transmission. If the message contains a Myo-specific pose, it triggers transitions between Finite State Machines (FSM) states, described in detail in Section 3.6, used to implement the different UPIs to control the prosthetic device. Suppose the desired action is to alter the current selection on the screen. In that case, a notification via another UART channel is sent to the independent μLCD’s microcontroller to perform the change it was ordered to and, thus, present visual feedback to the user. On the other hand, if the message contains raw EMG signals, the device fills up two circular buffers of signals collected by the electrodes placed near the palmaris longus and the extensor digitorum muscles (for unilateral below-elbow disarticulations). This way, customized methods to interpret the user intention can be used to adapt the bracelet to the prosthesis, such as works presented in [26,44].

### 3.3. Control Strategy

**Figure 3:**
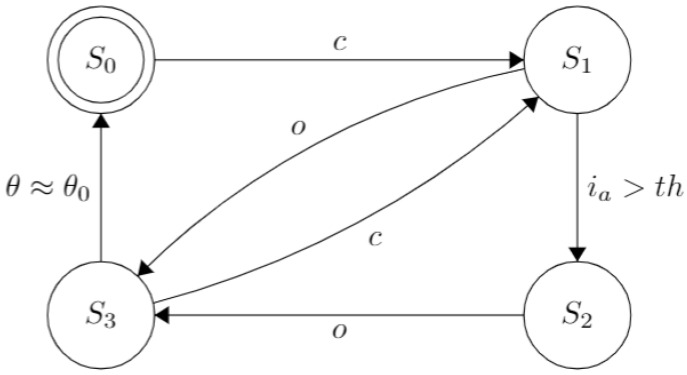
Finite State Machine demonstrating the opening/closing behavior of each finger on the prosthesis. $S_{0}$ indicates that the finger is entirely open; $S_{1}$, represents the flexion process triggered by the command *c*; $S_{2}$, indicates the finger is completely open (since $i_{a}>th$). Additionally, $S_{3}$ represents the extension process triggered by command *o* until $\theta \approx \theta_{0}$.

Once the user’s intent has been received, the high-level controller (HLC) uses this information to perform the necessary action that each finger must take to achieve predefined gestures and grips available to the user. Whereas at a low level, each finger functions with an individual hybrid control strategy for the flexion and extension processes, except for the thumb, which also has a quadrature encoder to implement a PI position controller to perform its rotation. Since the armature current $i_{a}$ of each DC motor is the only feedback signal measured from the system, a simple current on-off controller is implemented to perform the flexion process. In addition, a robust full-state observer is utilized to estimate the angular velocity and displacement, θ, of the gearhead shaft of each motor [42]. Thus, a robust state feedback controller is used to perform the extension process. This way, the prosthesis can perform the different predefined grasps, i.e., power and lateral grips, hook, etc. The functionality for each digit is illustrated in the Finite State Machine in Figure 3.

The prosthesis starts with all its fingers fully extended (in an “open” or “rest” position, at $\theta \approx \theta_{0}$), represented by the state $S_{0}$. Thus, when the command to move a particular finger, *c*, is received from the high-level controller, the transition to the state $S_{1}$ happens, activating the motor and causing the finger’s flexion. In this state, the RMS value of the armature current, $i_{a}$, is monitored continuously and, when a predefined threshold related experimentally to the fingertip wrench, th, is exceeded, the transition to $S_{2}$ happens. This parameter differs for each finger since each has discrepant mechanical factors due to their different size and length of the strings and elastics. Therefore, a proper calibration was made experimentally.

The finger is considered fully closed at this state and will start with the flexion process opening the finger if the *o* command is issued by the HLC, as shown by the transition from states $S_{2}$ to $S_{3}$. Finally, the transition from states $S_{3}$ to $S_{0}$ happens after the angular displacement, θ is approximated to its initial value $\theta_{0}=0$. This strategy was adopted since the elastic installed on each finger opposes itself to the coiling process but favors the unfurling one; therefore, ensuring that the motor shaft’s angular displacement is equal during both processes is essential. Finally, it is relevant to note that the closing/opening procedures may be interrupted and reversed if the appropriate commands are received.

### 3.4. Gestures Adapted to the Prosthesis

**Figure 4:**
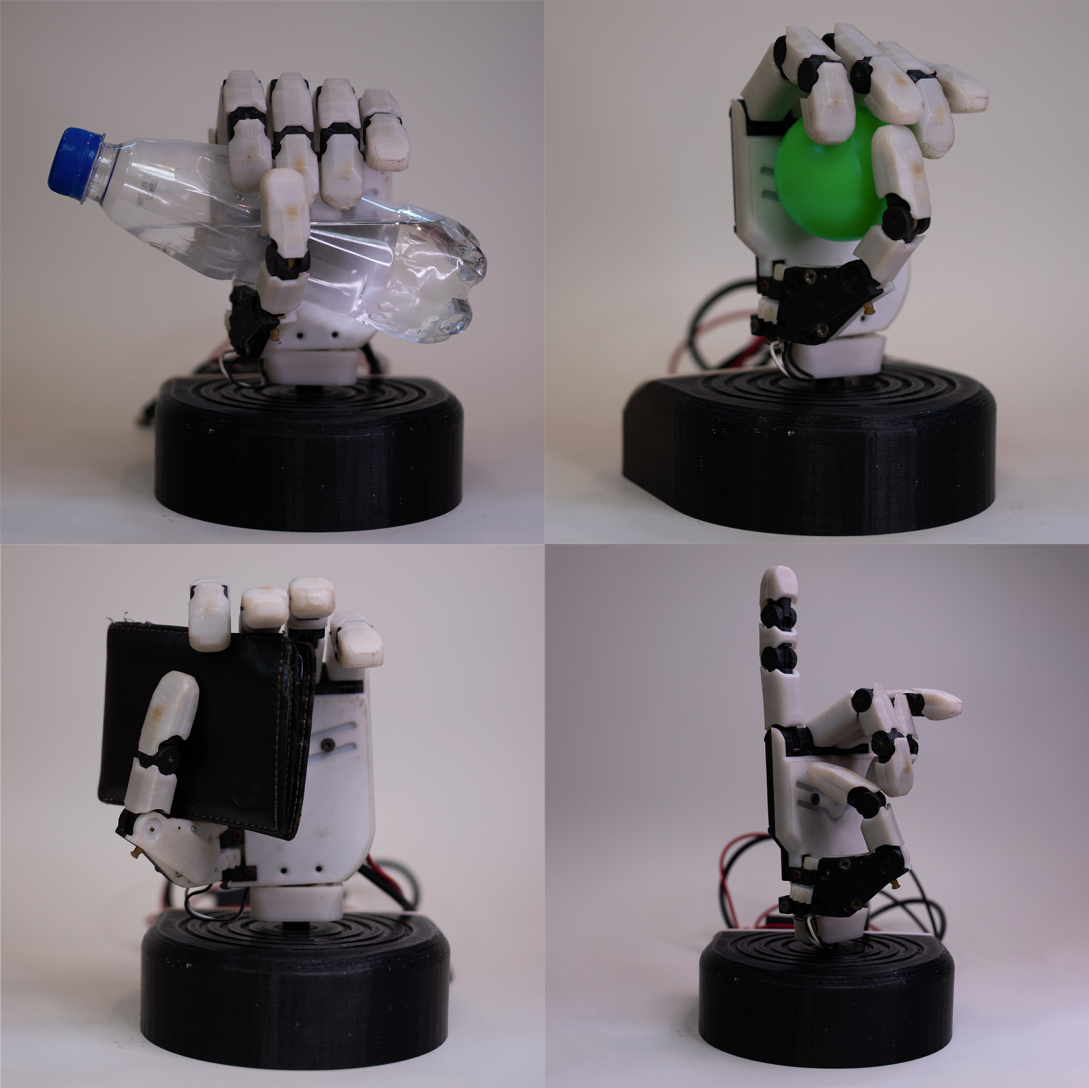
The image shows the Galileo Hand grabbing the objects used in the trials. On the upper left the hand is holding a ”water bottle”; on its right, a small plastic ”ball”; underneath, from left to right, holding a ”wallet” and ”pointing”, respectively.

The purpose of this subsection is to detail and clarify the actions at the patients’ disposal. The selected grasps are the following: “Close” (flexion of all the fingers and rotation of the thumb, power grasp), “Hook” (the thumb is the only finger extended, it is also adducted), “Lateral” (coiling of the strings of all fingers and the thumb is abducted), “Pinch” (flexion of the index and thumb, plus abduction of the thumb, precision grasp), “Point” (all motors are actuated, except for the index), “Peace” (all fingers are closed, except for the index and the middle finger), “Rock” (flexion of all fingers, but the index and the little finger; thumb adducted), “Aloha” (the index, middle and annular fingers are flexed), “Three” (all motors are actuated except for the index, middle and annular fingers), “Four” (similar to the previous gesture, but with the little finger extended), “Fancy” (the only extended finger is the little finger, with an adducted thumb) and “Index” (where the only flexed finger is the one giving the name to the action). Some of these gestures are illustrated in Figure 4. An important note is that some of the actions installed are for demonstrative purposes only. Other grasps may substitute some of the gestures for a more personalized approach or even reduce the number of actions available if they are not needed.

Now, the supported gestures for each evaluated interface will be enumerated. The traditional pattern recognition interface can complete the first four actions from the previous list. On the other hand, the version in Section 3.6.3, the one using the app, can fulfill the same as the previous iteration, plus “Pinch” and “Peace”. Finally, the rest of the interfaces allow the user to select any hand actions available on the menu.

### 3.5. NASA Task Load Index

The NASA-TLX test was used to measure and analyze the workload evoked by each interface under evaluation, as done in [11,19,22,40]. This test was selected to evaluate the impact that each UPI has on the users’ workload effectively. So, considering that the post-test evaluation techniques, such as SUS, do not permit evaluating different parts of the interface separately, and methods such as SEQ do not consider many different categories during testing, providing more binary results, the NASA-TLX scale was selected because it requires user testing through a post-task evaluation method for each interface taking into account six different workload categories: mental, physical, and time demand, the performance, the effort needed to operate it, and the frustration evoked. In this work, the index quantifies the effectiveness and performance of the workload to operate a prosthetic device using a given UPI; besides, it is also considered a more comprehensive test to evaluate user interaction, with well-known research and industry benchmarks to interpret scores in the context, which can be helpful for future works.

In addition, a binary response survey was used to determine if a user perceived an interface as user-friendly or not, intending to compare its results with the workload evoked by each UPI. Finally, a multifactorial ANOVA analysis is performed to determine how user-friendly an interface is according to the results obtained from the tests.

### 3.6. Experiment Design

Several interfaces were chosen for evaluation to determine the most relevant aspects for user-friendly interaction, affecting the workload of UPIs. The selection process was carried out by analyzing different interaction processes and considering the physical characteristics that correspond to traditional UPIs solutions; similar price ranges were also considered. Thus, the same one was adapted to work with each UPI to avoid selecting hardware bias to conduct the experiments. The different UPIs evaluated for this work are described hereunder.

#### 3.6.1. Multimodal Approach Using Buttons and Myo Interface

**Figure 5:**
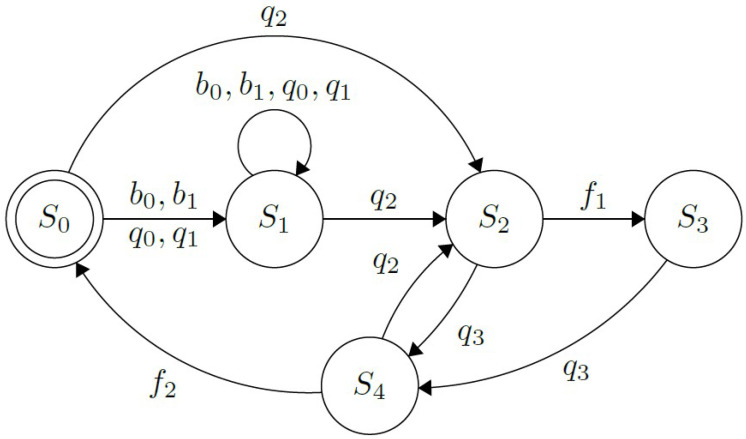
Finite State Machine showing the behavior of the interface using buttons and the Myo to operate. $S_{0}$ indicates that the hand is completely open; $S_{1}$, that there was a change in the selected grip; $S_{2}$, that the selected grip is being performed (when it is completed, the flag $f_{1}$ is lifted). In addition, $S_{3}$ represents that the hand is currently enacting the chosen gesture; while, $S_{4}$, that the fingers are opening (process that informs it is finished by lifting the flag $f_{2}$).

**Figure 6:**
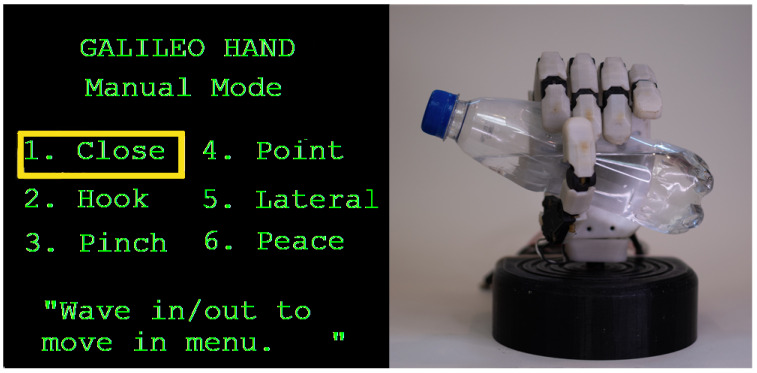
Galileo Hand’s graphical menu (**left**) and the prosthesis performing the action “Close” (**right**).

Based on the work presented in [18], this interface operates either by receiving gestures from the Myo armband or push buttons installed on the hand’s dorsal side to select a grip from the graphical menu or to perform an action. The functionality of this UPI is shown in the FSM in Figure 5. Both, the buttons, $B=\{b_{0},b_{1}\}$, and the muscle contractions subset, $Q=\{q_{0},q_{1},q_{2},q_{3}\}$, corresponding to Thalmic Labs’ “Myo poses”, are used to operate the prosthesis. By performing “wave out”, $q_{0}$, and “wave in”, $q_{1}$, hand extension and flexion respectively, as well as $b_{0}$ and $b_{1}$, causes a forwards or backward switch of the selected element in the menu displayed on the screen (shown in Figure 6); this process is represented by the state $S_{1}$. Besides, $S_{0}$ indicates that the fingers on the prosthesis are fully extended, in their default initial state; while in $S_{3}$, the hand is currently performing the chosen grip. An important aspect to note is that, whilst in this state, changing the menu’s selection is presented to the user, as the motor activation processes’ timing differs between actions and could lead to wrong finger positioning if the case arose.

Furthermore, $S_{2}$ and $S_{4}$ indicate that the prosthetic device is currently closing or opening its fingers, respectively. These procedures can be interrupted by each other if a correct command is received. In addition to that, to execute an action $q_{2}$, “fist” needs to be performed by the user. At the same time, both “double tap” (two swift, consecutive contractions) and “fingers spread” are the contractions $q_{3}$ that deactivate the action. It was decided to use both gestures to deactivate the user’s selected actions according to the results shown in Section 4. Finally, the last elements in the FSM representing the interface’s behavior are the flags $f_{1}$ and $f_{2}$. The first one is triggered when all the fingers have reached their desired position when performing an action, while the second triggers when all the fingers returned to their initial position, $\theta_{0}$.

#### 3.6.2. Myo-Powered Interface with a Reduced Contractions Subset

**Figure 7:**
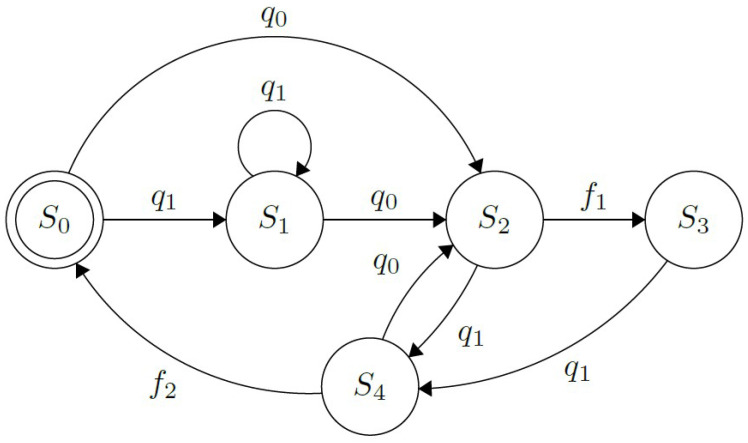
Finite State Machine representing the UPI interaction process from the version with the reduced contraction subset. $S_{0}$ indicates that the hand is completely open; $S_{1}$, that there was a change in the selected grip; $S_{2}$, that the selected grip is being performed (when it is completed, the flag $f_{1}$ is lifted). In addition, $S_{3}$ represents that the hand is currently enacting the chosen gesture; while, $S_{4}$, that the fingers are opening.

This interface works similarly to the multimodal one explained in Section 3.6.1, i.e., selecting the desired action in a menu and performing it with an “activation pose”. The main difference is that the subset, $Q=\{q_{0},q_{1}\}$, is reduced to only two contractions. In this way, it is imitating the iteration proposed in [22,42], by utilizing “wave in” to act and “wave out” to select and deactivate a grip, illustrated in Figure 7. This simplified subset provides a viable alternative if some of the Myo poses are unperformable by the patient. Additionally, the buttons are absent for this UPI to help accommodate a reliable solution to bilateral amputees.

#### 3.6.3. Multimodal Approach Based on Object Classification and Detection

This version uses a mobile application to control the prosthesis. The device possesses a camera facing the palm, which takes pictures of the objects to be interacted with and suggests a grasp. Alternatively, the photos can be taken with the mobile device’s photographic equipment. By performing Myo’s poses, the user can either accept, reject or cancel the recommended grips provided by the app’s detection algorithm. This process uses a bag of words computer vision algorithm to assign a label to the detected object with a grip. This is a replica of the one used in [45].

**Figure 8:**
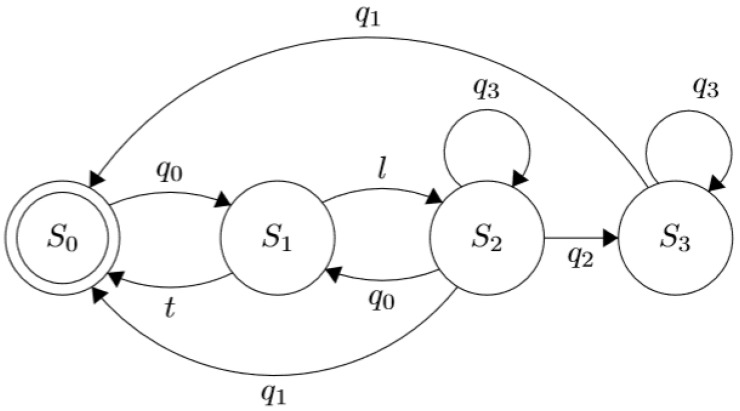
Comportment of the UPI from the version based on object recognition. $S_{0}$ indicates that the prosthesis it is completely open; $S_{1}$, that a picture is being taken; $S_{2}$, that a label is being determined (when this process is finished, the flag *l* is lifted, if not, timeout *t* is raised); and $S_{3}$, that the action is being executed.

**Figure 9:**
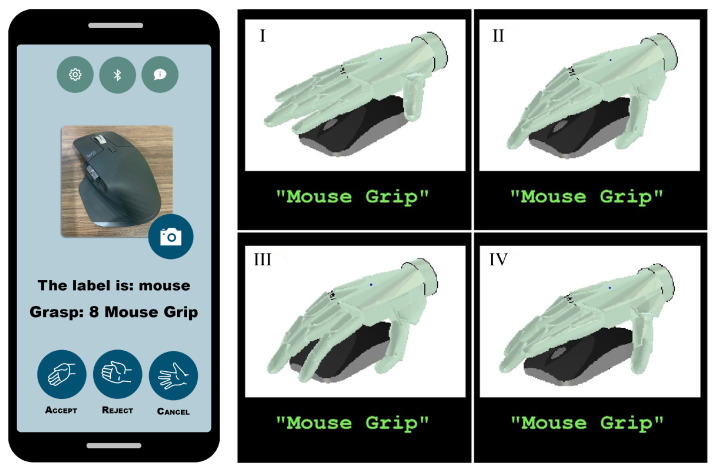
On the left, the visual feedback presented to the user on the Android app. Beside it is the animation of the grip, which is shown to the user via the Galileo Hand’s LCD screen.

The interface’s behavior is described as shown in Figure 8, where the set of contractions, $Q=\{q_{0},q_{1},q_{2},q_{3}\}$, represent the Myo poses which are used to choose along with the states of the FSM: “fist”, “fingers spread”, “wave in” and “‘wave out”, respectively. The interface’s behavior is described as shown in Figure 8, where the set of contractions, $Q=\{q_{0},q_{1},q_{2},q_{3}\}$, represent the Myo poses which are used to choose along with the states of the FSM: “fist”, “fingers spread”, “wave in” and “‘wave out”, respectively. The state $S_{0}$ denotes that the prosthetic device is in its rest position with all its finger entirely open. Simultaneously, the UPI stays idle until the user performs the contraction $q_{0}$ to trigger a transition to the state $S_{1}$ where the system takes a picture of the object with which he wants to interact, and then is classified by the CNN algorithm running in a smartphone until a valid label *l* is defined. Thus, the label is validated when the classification certainty reaches a heuristic threshold that triggers the transition to the state $S_{2}$. If the CNN classification does not return a valid label, the system returns to the initial state $S_{0}$, upon a predefined timeout *t*. In the same state, $S_{2}$, when $q_{1}$ is performed, the transition indicates that another photo needs to be taken, canceling the action selection process. The contraction $q_{2}$ accepts the algorithm’s suggestion while $q_{3}$ rejects it, so the system proposes another grasp or gesture. The text and animations of the suggested grip are provided as visual feedback via the LCD screen, as shown in Figure 9.

#### 3.6.4. sEMG Pattern Recognition

Based on [17], this interface consists of a system that, utilizing Myos’s pattern recognition methods, maps each of the predefined “Myo poses” to a grip to be performed. So, the prosthesis executes an action after receiving the interpreted contraction from the armband.

The layout is defined as follows: “fist” and “fingers spread” to close and open all the fingers, respectively; “wave in” to a pointing position; “wave out” to carry out a lateral grasp; and “double tap” to a hooking stance. The gestures were selected according to their usability in ADLs, an aspect that was also taken into account when assigning the actions concerning Myo’s success rate.

## 4. Results and Discussion

### 4.1. Myo Armband Efficiency

The myoelectric classifier embedded in the Myo armband is not fault-free; some contractions are misclassified at times, even for people without muscle damage. Therefore, a confusion matrix was elaborated to corroborate the results shown in works such as [24] and to verify its reliability in gathering the user intention. This analysis also served to select which of the Myo armband-supported poses are the most adequate to be implemented as default contractions to operate each interface. Therefore, depending on the amputation level, the Myo will not correctly classify all contractions for limb-impaired subjects.

**Figure 10:**
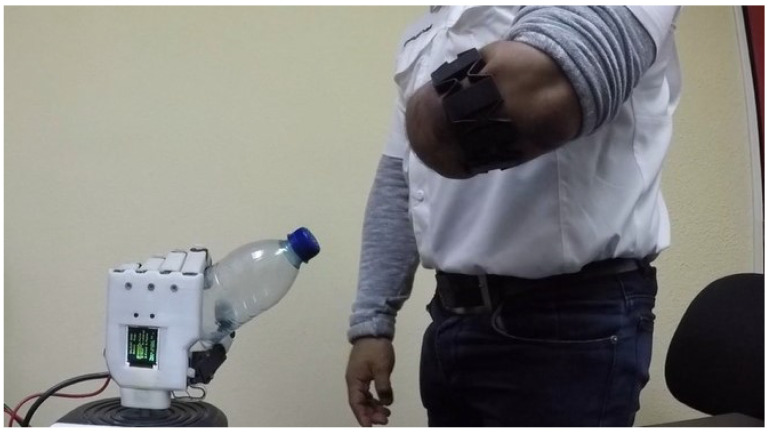
A limb-impaired volunteer testing the UPI with the reduced contraction set of muscles.

The data was obtained in two stages, one for the able-bodied subjects, done in [22], and another for the unilateral limb-impaired ones (as depicted in Figure 10). The first was composed of 8 males and 2 females between the ages of 22 and 35, while the latter, by 2 male volunteers of 30 and 55 years old, as shown in Table 1.

**Figure 11:**
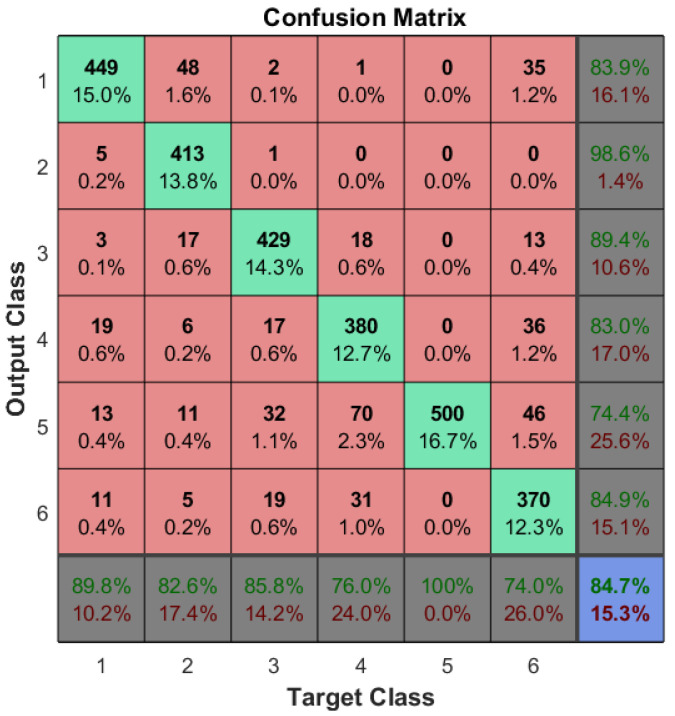
Confusion matrix evaluating the default classifier of the Myo.

To avoid biased results, these volunteers had no experience whatsoever with the Myo armband. Even though a more comprehensive range of ages may provide more accuracy to a generalized population, the musculature differences tend to be minimal, as the amputation damages it in a similar manner [27]. So, able-bodied subjects were asked to perform every Myo pose in its default roster 50 times; while noting what the classifier detected each time. The resulting matrix is shown in Figure 11, where the default MYO poses are numbered as follows: (1) “wave out”, (2) “wave in”, (3) “fist”, (4) “double-tap”, (5) indicates a no-operation (NOP) meaning the armband did not detect any pose and (6) “fingers spread”. According to the results gathered by this experiment, the Myo poses were mapped to the operation actions in diverse manners to the different interfaces. These results do not include the tests from the two impaired volunteers due to the poor accuracy obtained with some contractions performed by non-disabled people (specifically, “fingers spread” and “double-tap”). This was also reflected in the constant misclassification of these contractions from the limb-impaired ones. Therefore, the data gathered from this type of volunteers was merely regarding the interface that obtained the closest overall performance compared to the UPI described in Section 3.6.4, according to the able-bodied subjects’ results.

The total accuracy achieved by the default classifier of the bracelet was about 87.7%. As was expected, NOP was always classified correctly. On the other hand, as shown in Figure 11, three gestures (“wave in”, “wave out” and “fist”) reached acceptable performance metrics in terms of accuracy (diagonal cells), precision (the column on the far right), and recall (the row at the bottom). In this way, for the interface employing a multimodal approach using the MYO bracelet in tandem with buttons, “wave in/out” were selected to naturally choose between a set of predefined gestures and “fist” activates the selected gesture on the prosthetic device. In this way, for the interface employing a multimodal approach using the MYO bracelet in tandem with buttons, “wave in/out” were selected to choose between a set of predefined gestures naturally. At the same time, “fist” activates the selected gesture on the prosthetic device. The remaining gestures, the ones with the least successful rates (“finger spread” and “double-tap”), were selected to return the prosthesis to the rest position. Thus, the high error rate of these gestures cannot influence the UPI’s performance since the user cannot select or change a gesture while the prosthesis is acting.

Moreover, the UPI that employs deep learning-based artificial vision algorithms was replicated precisely from work proposed in [45]. Since this approach utilizes four muscle contractions to operate the interface, the same (“wave in”, “wave out”, “fist” and “finger spread”) were elected to interact with prosthesis and the android mobile application. Thus, the gesture with minor performance metrics was elected to deactivate the prosthesis, returning the fingers to the rest position. Finally, for the UPI based on sEMG pattern recognition (Section 3.6.4), the contractions with the most significant performance rates were mapped to the most useful grips according to the user’s preferred ADLs. However, it is considered a natural mapping that facilitates the operation of the prosthetic device, where “fist” activates the power grip action, “fingers spread” open the prosthesis, “wave in” could be used for customized grips and “double tap” for a least used gesture.

Regarding the UPI based on a reduced set of contractions (Section 3.6.2), the set of contractions was selected, taking into account the contractions that ended with better performance (accuracy, precision, and recall). In this way, “wave in” was selected to activate predefined grips and gestures, while “wave out” was chosen to select between the different predefined grips and gestures and also to return to the open position. Thus, in this iteration, the system avoids using the actions with low success rates and replacing them with the most accurate ones, ensuring better performance for the limb-impaired ones, as well as increasing the functionality of the prosthetic device by performing only these contractions. This alternative is possible since the menu is blocked during a gesture’s performance, so both hand extension and flexion are available to return the hand to its default state.

### 4.2. NASA Task Load Index Evaluation

The first evaluation process consisted of asking the non-disabled volunteers to rate each of the UPIs mentioned above on each category on a scale divided into 20 intervals, with a lower score indicating a better result. The test consisted of performing different gestures and utilizing different grasps to interact with commonly encountered everyday objects. The trials were held after providing the subjects a training period (a couple of minutes) for them to be accustomed to the interfaces (as indicated by each participant); this was to avoid any bias regarding the order in which the interfaces were tested, which was done randomly, not unlike [27]. The actions asked to be performed by the volunteers were: to hold a small plastic ball, a water bottle, and a wallet, as well as to press a specific key on a laptop’s keyboard. These were selected for them among the most common grasps in ADLs that correspond to Cutkosky grasp taxonomy [46]. The tasks, as mentioned earlier, were repeated thrice so that the subjects could adequately adapt to each operational mode. Additionally, the performances of these actions can be easily evaluated, as one can visualize the output of the keyboard on a computer screen, and the grips should hold the objects firmly. In addition, since the purpose of this study is to evaluate the workload of the user interface only, the terminal device was not attached to the volunteers’ limb. In this way, both the weight of the prosthesis and the objects do not directly influence the physical demand evoked by each user-prosthesis interface.

**Figure 12:**
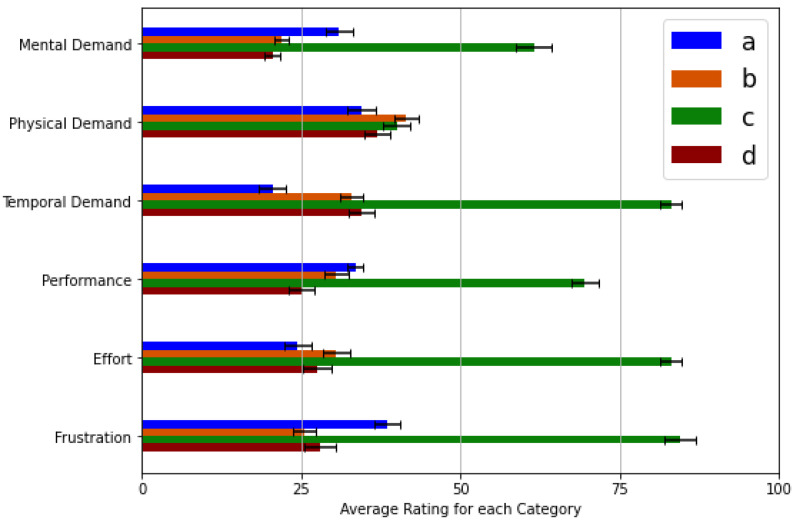
Mean of the results gathered from the volunteers. Where (**a**) is the sEMG PR UPI; (**b**), the one using the buttons and the Myo; (**c**) is the version using the camera; and (**d**) is the iteration with reduced contractions subset.

**Figure 13:**
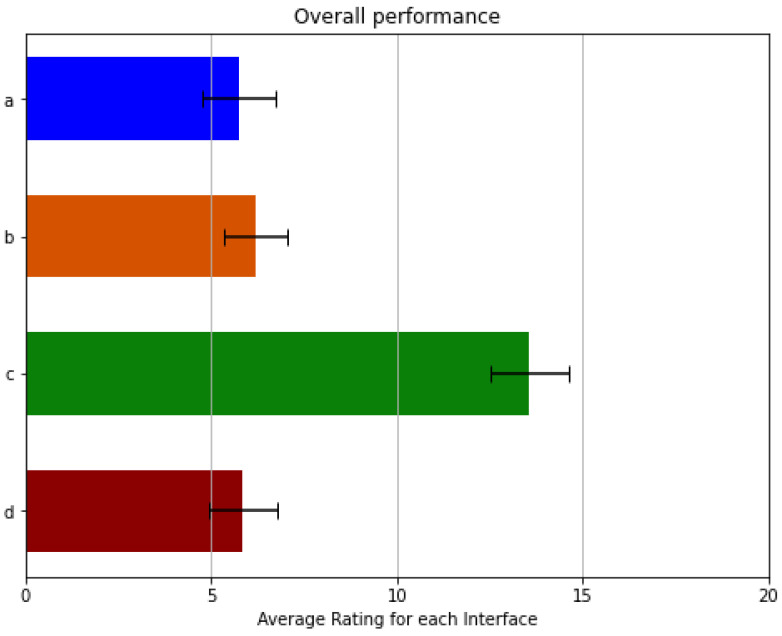
Overall performance of the different versions. (**a**) is the sEMG PR iteration; (**b**) is the one with the buttons; (**c**) uses the computer vision algorithms; and (**d**) is the interface utilizing a reduced contractions subset.

This assessment was carried out with the same volunteers as the previous experiment. Considering that not every workload class carries the same level of relevancy in the prosthetic field, these preliminary results may show bias or skewness if not appropriately weighted. Thus, an overall performance statistic was determined Figure 13 which calculates a weighted average of all categories for each interface, ranking them based on feedback from the volunteers, opinions of expert engineers, and remarks from several patients, in this order (from most important to least): Temporal Demand, Mental Demand, Physical Demand, Performance, Effort & Frustration. Figure 12, shows the means and the standard deviations for each of the considered categories. The results reflect a significant discrepancy between the UPI that uses deep learning-based computer vision algorithms and all other UPIs, showing an inferior interface that presents a significant workload in several categories. Therefore, performing a Factorial Analysis of Variance (ANOVA) test on the results obtained demonstrates a significant difference in contrast to the UPI described in Section 3.6.2. In addition, with a critical value of 3.84 and an alpha of 0.05, the F statistic obtained for this test was about 132.4. This value discards the main effect hypothesis, showing a significant inequality between the evaluated interfaces.

The interface based on sEMG pattern recognition presents the best results in physical and temporal demand categories and on the category that evaluates the user’s effort to complete a task. Furthermore, the multimodal UPI that employs buttons in tandem with the Myo bracelet resulted in the least frustrating interface for users. In contrast, the UPI based on reduced contractions subset obtained better results than the others in the performance and mental demand categories. All three interfaces proved to be proficient in the different categories; however, the results (as shown in Figure 12) do not show a significant difference to determine which of them has a better overall performance. These results showed that all interfaces are straightforward iterations with an overall performance around the upper 70% according to the NASA TLX’s scale. The obtained means for the remaining UPIs are still pretty similar. As shown in Figure 13, the UPI (a) has a mean of 5.75; (b) one of 6.2; and (d) 5.86, Therefore, more Factorial ANOVA analyses were performed on these interfaces with the same alpha value. All previous tests were performed comparing the reduced contractions subset version to the other interfaces to corroborate improvements or significant differences due to several participants’ interest in an alternative to a PR-based UPI. Thus, these results show that the different aspects involved in the interaction process do not affect the workload in a relevant matter.

**Figure 14:**
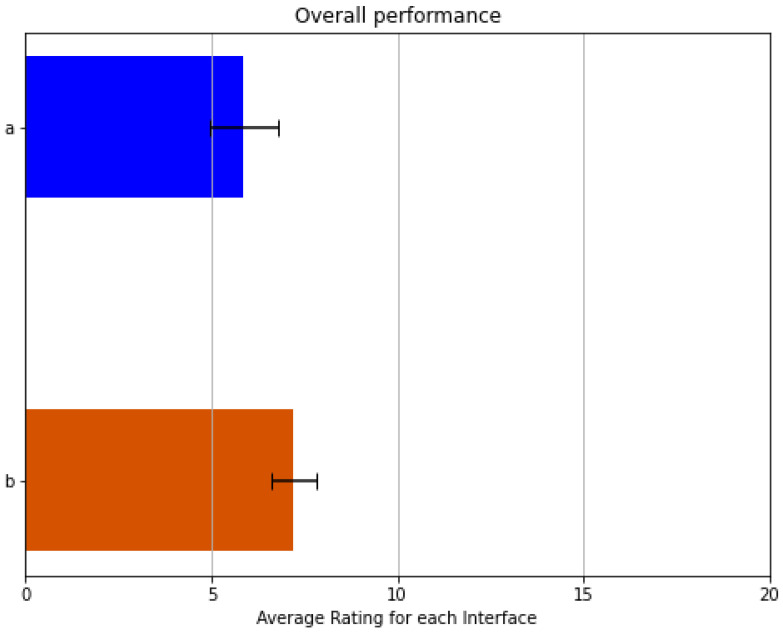
Overall performance of the reduced contractions subset version. (**a**) is the score from the able-bodied subjects; and (**b**) is the one with volunteers with upper-limb difference.

The second evaluation process consisted of requesting the limb-impaired subjects to perform the same ADLs from the preliminary testing by using the reduced contractions subset UPI. This way, one can compare the performance concerning the other volunteers’ quantified results. This new score averaged 7.2 in the TLX scale (with 2.39 standard deviation), as shown in Figure 14. Compared to the average value of able-bodied subjects for the same interface (5.86), an ANOVA test was performed, and the results show no significant difference between groups. The F-statistic obtained was 0.78, a critical value of 3.98 with an alpha value of 0.05. Additionally, every volunteer sent a survey to determine if the interfaces are considered user-friendly. The PR, the multimodal approach, and the reduced contractions subset interfaces show an acceptable result, as around 70%, 80%, and 90% of participants perceived them to be user-friendly UPIs, respectively. On the other hand, the only UPI-based that shows poor results was the one based on object classification and detection since only 30% of participants perceived it as user-friendly.

## 5. Conclusions

An effective interaction process between the user with the prosthesis is a very relevant aspect that users consider when selecting an assistive device and thus continue to use in their ADLs. Therefore, it is essential to identify the aspects favoring or opposing the target users when designing a more efficient and user-friendly interface. The results for the interface described in Section 3.6.3 showed a trend strongly tied between the execution time of the actions and their subjective evaluation, as evidenced by the poor reception and the long operation time required to select and execute an action on the prosthetic device. These strongly impact the process of interaction with the most common objects that are part of its environment. This perception on users can be caused by the amount of time it takes to select the object with which the user wants to interact and then take a photo of it that must be processed to suggest the proper grip or gesture. Thus, this process becomes complex and tedious for users, evoking frustration and demanding more effort to achieve a particular goal. In addition to that, if the system employs the camera mounted on a mobile device (s.a. smartphones or tablets), the user requires an able-bodied hand to operate it with the app, needing particular physical prowess not possessed by certain kinds of patients, specifically by bilateral amputees. If the system uses a camera mounted on the prosthetic device, the weight and position of the camera can influence the effectiveness of the UPI since it is crucial for the system to frame the object with which the user wants to interact appropriately. Moreover, the object classification and detection algorithms impose another requirement to the system in terms of the processing device’s performance running the interface’s software. This increases the price, either by the need for a smartphone or an embedded system that is powerful enough to run the necessary machine learning methods. Since these accommodations are not easily attainable in developing countries due to the general shortage of high-speed internet, cellphone service, or even electricity, these restrictions mainly affect amputees from regions suffering from poverty. In this way, this iteration was the worst evaluated both in the survey and in the NASA-TLX test, demonstrating that multimodal alternatives do not always improve the interaction between the user and the assistive device, especially when the interaction process becomes very complicated for the user.

Regarding the results showed in Figure 12, the superiority of the interface presented in Section 3.6.4 lies in the swift selection of grips and gestures. This perception is due to the lack of a menu with which it is necessary to select the desired action. Therefore, the results obtained on the physical demand and the required effort categories are low. In contrast, the results for frustration and mental demand for this iteration are caused by the need to memorize which Myo contractions activate a predefined action, resulting in a slightly more complex process for patients. This is also frustrating for the limb-impaired subjects since customized pattern recognition systems (requiring extended periods of training) are needed to achieve low misclassification rates, and still, only a limited number of actions can be selected. However, these impressions show that no visual feedback is necessary for a UPI to be user-friendly, leading to a simpler and more affordable alternative as long as the user can still operate the prosthesis. For these reasons, this interface was the third-best evaluated by the volunteers, despite the good results obtained from the NASA-TLX test, which show that the workload is relatively low for this iteration. On the other hand, this interface is the one that seems to interact with the device more naturally. However, technological advances are still needed in biomedical signal processing and pattern recognition to naturally interpret the user’s intention, especially using affordable approaches available to amputees.

Furthermore, the results also show a lack of frustration for the UPI presented in Section 3.6.1, being the second-best evaluated by volunteers. This perception may result from the sporadic inexactitude of the default Myo classification process. This UPI provides an alternative to navigate along with the menu by using buttons; therefore, an EMG classifier is not strictly necessary to select an action but to confirm it, which provides a satisfactory alternative in a multi-modal approach. This leads to the fact that a pattern recognition system may not be necessary, which vastly reduces training time and the complexity of the EMG sensor and the device gathering the user intent. This allows for a simpler and less expensive solution for amputees, as only two sEMG channels in combination with traditional digital signal processing techniques are required to detect muscle activity from both flexor and extensor sets of muscles [23]. This is especially valid considering that volunteers stated that they only need different grips to hold various types of objects, not an extensive array of hand actions, meaning that the contractions to be assessed do not need to be vast, allowing for a more straightforward and intuitive interface. However, a UPI involving mechanical interaction (i.e., pressing buttons) is not a feasible solution for bilateral amputees, as the interaction process does not favor them.

Furthermore, the results also show that mental exertion needed to operate the best evaluated UPI described in Section 3.6.2 achieve the lowest score on the scale. This perception from the volunteers may occur since the user does not need to memorize the particular mapping that relates a contraction with a grip or gesture, nor do they need to consider using the buttons installed on the top of the artificial limb. Since the subset of contractions for this UPI is limited (only two contractions), the mental demand is also reduced because the contractions were carefully selected to operate the device naturally. Besides, the performance for this interface results in being the best along with all the interfaces. This advantage may be due to the accuracy with which the Myo interprets the pose used to return the prosthesis to its rest position compared to its multimodal counterpart. The frustration level also scores low, particularly on unilateral amputees, which may be due to their ability and experience to adapt their ADLs to employ one healthy hand with the help of an assistive device. Thus, such patients do not need many complex grasps, as they prefer to carry out the mechanically dexterous tasks with their undamaged limbs. A typical example is opening a bottle, which may be easily done by holding it firmly with the prosthesis and turning the cap with the other hand. Nevertheless, bilateral amputees are not benefited from such a reduced pool of alternatives. However, another advantage of this version over the PR one, though not explicitly shown on the overall scores, is that a broader range of actions might be provided without the need to increase the number of contractions detected.

On the other hand, after conducting these trials, the multimodal approach using a mechanical input (buttons) and the one based on reduced contractions set did not result in a relevant improvement. The same conclusion can be drawn to the UPI that employs an extended subset of contractions and a range of actions. These experiments demonstrate that a simpler and more affordable UPI results in a similar interface to the user. However, reducing the contractions subset to operate the device can restrict the operation mode to fit each amputee’s unique necessities, prompting the user to employ the prosthesis even if they are unable or unwilling to complete certain Myo’s poses. In addition, these results could vary due to the lack of evaluation by bilateral amputees in this study.

The results collected during this research give us a better idea of how different approaches used to interact with upper limb prostheses affect the user’s workload and interface amiability. This can be used to find alternatives to improve the price, performance, reception, and adaptation of such assistive devices by reducing the workload required to operate them and the interaction process’s complexity altogether. This leads to believe that the UPI does not need to be a complex one, as shown by the results for the one using the camera, but a simple, functional one, preferably using the smallest contraction subset possible (to increase the range of users able to operate it). The time required to complete a grasp was also shown to be an essential aspect when evaluating the interfaces, which is unsurprising considering it may be compared to the response time of the healthy limb. Finally, even though there is a substantial difference between able-bodied and limb-impaired subjects, this research work’s results do not show a significant deviation, as the tests averaged a similar score, and most discrepancy comes from variance within groups instead of between groups. Therefore, the evaluation process using only healthy subjects benefits the user-friendly UPI design process. Thus, it can help the UPIs designer discard or favor possible solutions before being tested by people suffering from upper limb amputation according to the analysis of the evaluation results and then test only the best iterations. It is best to test selected iterations for deeper analysis regarding an interface’s evoked workload and amiability for this kind of volunteer. This way, we can provide better UPIs that will improve the quality of life of those who need it.

## Figures and Tables

**Table 1:** List of volunteers used in the experiment.

No.	Limb-Impaired	Prosthesis User?	Age	Gender
1	No	No	25	M
2	No	No	27	M
3	No	No	24	M
4	No	No	24	F
5	No	No	23	F
6	No	No	23	M
7	No	No	23	M
8	No	No	26	M
9	No	No	22	M
10	No	No	35	M
11	Yes	Yes	55	M
12	Yes	No	30	M

## Data Availability

Datasets and original images are available from the corresponding author on request.
